# Direct measurement of Tan’s contact in a one-dimensional Lieb-Liniger gas

**DOI:** 10.1126/sciadv.adv3727

**Published:** 2025-10-03

**Authors:** Qi Huang, Hepeng Yao, Xuzong Chen, Laurent Sanchez-Palencia

**Affiliations:** ^1^ Institute of Quantum Electronics, School of Electronics, Peking University, No. 5 Yiheyuan Road, Haidian District, Beijing 100871, China.; ^2^ International Center for Quantum Materials, School of Physics, Peking University, No. 5 Yiheyuan Road, Haidian District, Beijing 100871, China.; ^3^ DQMP, University of Geneva, 24 Quai Ernest-Ansermet, Geneva CH-1211, Switzerland.; ^4^ CPHT, CNRS, Ecole Polytechnique, IP Paris, 91120 Palaiseau, France.

## Abstract

The Tan contact is a pivotal quantity for characterizing many-body quantum systems, bridging microscopic correlations to macroscopic thermodynamic behavior. It is defined as the weight of universal $1\;/\;k^{4}$ momentum tails, but, so far, its direct measurement has been hindered in Bose gases due to interactions strongly altering time-of-flight imaging. Here, we report the first direct measurement of the Tan contact in a strongly correlated Lieb-Liniger gas. Leveraging the one-dimensional geometry of our system, we implement a two-stage expansion scheme, yielding interaction-immune imaging. Our results show good agreement with theoretical predictions and are consistent with a predicted universal scaling law. Our work paves the way for further characterization of the Lieb-Liniger gas across broad interaction regimes and holds promise for extension to other correlated quantum gases in confined geometries.

## Teaser

With a two-stage time-of-flight imaging, the direct measurement of the Tan contact in a one-dimensional quantum gas is reported.

## INTRODUCTION

Interactions play a pivotal role in determining the physical behavior of many-body systems and drive quantum phase transitions (*1*–*3*). The simplest model, which considers pairwise contact interactions, is sufficient to account for the main features of most many-body systems in condensed matter and strictly applies to most ultracold atomic gases, where the interaction strength can also be tuned via magnetic Feshbach resonances (*4*–*6*). Such contact interaction implies a zero-distance singularity of the many-body wave function, which manifests in momentum space as a characteristic momentum distribution with large-momentum tails scaling as $n\left(k\right)≃C/k^{4}$ (*7*–*9*). This universal behavior applies irrespective of the nature of particles, system dimensionality, temperature, and interaction strength. The constant *C*, known as the Tan contact, may be interpreted as a thermodynamic conjugate of the interaction strength (*9*, *10*) and is related to a number of quantities, including thermodynamic potentials, pressure, entropy, as well as interaction energy, and two-body correlations, within the so-called Tan relations (*11*, *12*). In one dimension (1D), recent theoretical work has shown that the Tan contact provides fruitful information about the specific effects of strong correlations, including the celebrated Tonks-Girardeau (TG) fermionization of strongly interacting Bose gases (*13*–*23*).

For most ultracold atomic gases, pairwise contact interaction is guaranteed by dilution and s-wave scattering at low energy (*4*, *5*), with noticeable exceptions for dipolar and Rydberg-atom gases. In recent years, several measurements of the Tan contact have been reported for interacting Fermi and Bose gases (*10*, *24*–*29*). Strongly interacting Fermi gases have allowed measuring the contact by various techniques (*24*, *25*, *28*, *29*) and verifying universal Tan’s relations (*24*). The Tan contact has also been measured for weakly interacting Bose gases in 2D and 3D, using radio frequency spectroscopy (*26*) and Ramsey interferometry (*10*). In contrast, exploration of the Tan contact for strongly interacting Bose gases, in particular in 1D, is still missing. So far, a major impediment to direct observation of universal $1\;/\;k^{4}$ momentum tails and direct measurement of the contact is that interactions during standard time of flight (TOF) strongly affect the momentum distribution (*30*). Moreover, impurities, as created by spin-flip processes in magnetic traps, have been shown to alter the expansion dynamics and produce dynamical $1\;/\;k^{4}$ tails with a strongly modified contact (*31*, *32*). Last, high temperatures may induce hole anomalies with $1\;/\;k^{3}$ contributions, which screen the $1\;/\;k^{4}$ tails (*33*).

In this work, we report the first observation of universal $1\;/\;k^{4}$ tails in the momentum distribution of a strongly interacting 1D Bose (Lieb-Liniger) gas and direct measurement of the Tan contact from their weight, overcoming these issues. Using a purely optical confinement and leveraging the 1D geometry, we realize a contact-preserving TOF measurement of the momentum distribution via a two-stage expansion scheme. The experimental data for the momentum distributions and the measured contacts show good agreement with quantum Monte Carlo (QMC) calculations for various values of temperature and particle number. Our results show clear beyond mean-field many-body effects and provide the first verification of the predicted universal two-parameter scaling of the contact for trapped, finite-temperature, Lieb-Liniger gases (*21*).

## RESULTS

### Emulating the trapped Lieb-Liniger model

The extended Lieb-Liniger model we consider is governed by the Hamiltonian

$$\hat{H}=\sum_{i}\left[-\frac{\hbar^{2}}{2m}\frac{\partial^{2}}{\partial x_{i}^{2}}+V\left(x_{i}\right)\right]+g_{1D}\sum_{i<j}\delta \left(x_{i}-x_{j}\right)$$ (1)

where ℏ is the reduced Planck constant, *m* is the atomic mass, $x_{i}$ is the position of particle *i*, and $V\left(x\right)=m\omega_{x}^{2}x^{2}/2$ is an external harmonic potential with frequency $\omega_{x}$ . The second sum accounts for point-like pairwise interactions, and the coupling constant may be related to the 1D scattering length $a_{1D}$ via the formula $g_{1D}=-2\hbar^{2}/{\mathrm{ma}}_{1D}$ (*34*, *35*). In ultracold atom systems, it is realized by strongly confining a 3D Bose gas to zero-point transverse oscillations using a strong transverse harmonic trap of frequency $\omega_{⊥}$ . The 1D scattering length $a_{1D}$ is then related to the 3D scattering length $a_{3D}$ and the transverse oscillation length $\ell_{⊥}=\sqrt{\hbar /m\omega_{⊥}}$ , via $a_{1D}=-\ell_{⊥}\left(\ell_{⊥}/a_{3D}-C_{0}\right)$ with $C_{0}=\;|\zeta \left(1/2\right)|/\sqrt{2}\approx 1.0326$ and ζ being the Riemann zeta function (*5*, *34*). For an homogeneous gas with 1D density $n_{1D}$ , the interaction regime is characterized by the Lieb-Liniger parameter $\gamma ={\mathrm{mg}}_{1D}/\hbar^{2}n_{1D}$ (*36*, *37*). For γ≪1 (high density), the system is weakly interacting and may be approximately described using mean-field theory. For stronger interactions, γ≫1 (low density), the Bose gas crosses over toward the TG regime, where the bosonic many-body wave function can be mapped onto that of free fermions. This effect is known as TG fermionization (*38*).

**Fig. 1.**
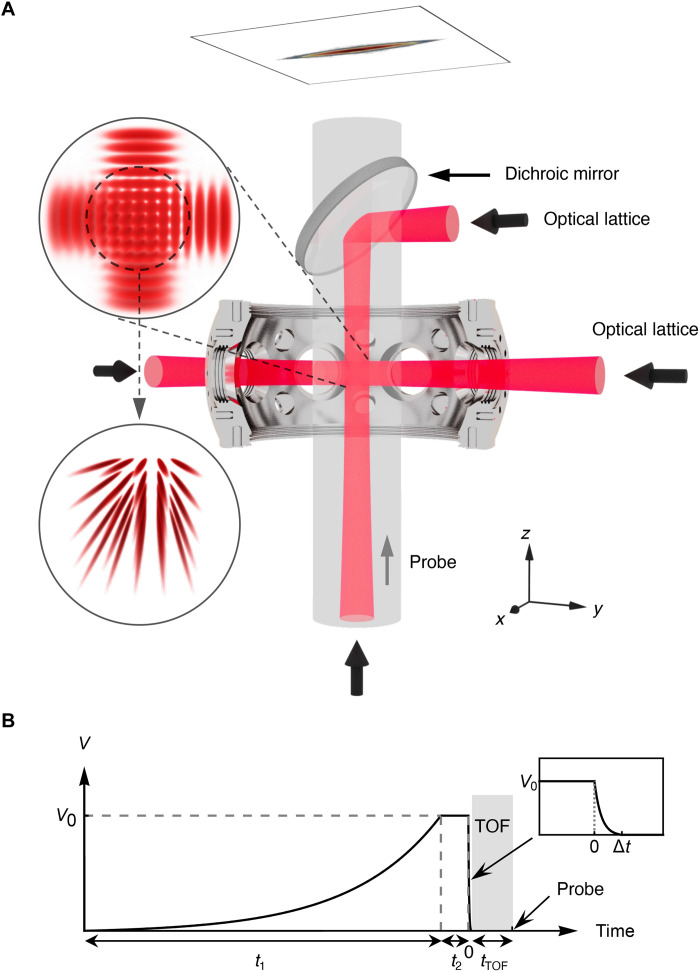
Sketch of the experimental setup and sequence. (**A**) An array of one-dimensional tubes are generated by a strong 2D lattice potential realized by the standing wave from two retroreflected laser beams along the *y* and *z* axes (red). The tubes are then formed along the *x* direction (insets), and imaging is performed along the *z* direction. (**B**) The 2D lattice is progressively ramped up to from 0 to *V*_0_ over a time *t*_1_ = 260 ms, and it is then held for a further *t*_2_ = 20 ms. TOF expansion is induced by ramping down the lattice beams to zero in Δ*t* = 50 μs and let to expand freely for another *t*_TOF_ = 30 ms.

In our experiment, we start with a 3D Bose-Einstein condensate (BEC) of ^87^Rb atoms confined in an optical dipole trap, with minimum temperature less than 20 nK. The atom number of the nearly pure BEC varies from 3 × 10^4^ to 1.5 × 10^5^, depending on the chosen experimental parameters. We then load the BEC into a strong 2D optical lattice formed by an orthogonal pair of retroreflected laser beams with wavelength 1064 nm in the *y* and *z* directions (Fig. 1A). The loading process is realized by ramping up exponentially the intensity of the optical lattice from 0 to *V*_0_ = 70 *E*_r_ in a time *t*_1_ = 260 ms (Fig. 1B). Here, $E_{r}=\hbar^{2}k_{L}^{2}/2m$ is the recoil energy with *k*_L_ the laser wave vector. The 2D optical lattice generates an array of independent, parallel 1D tubes orthogonal to the laser beams (see insets of Fig. 1A). For such a large laser intensity, tunnel coupling between the tubes is negligible, as evidenced by the absence of observable interference in 3D TOF imaging. We then hold the system for a further *t*_2_ = 20 ms and let it equilibrate. For a 2D lattice amplitude of *V*_0_ = 70 *E*_r_, the transverse confinement in each tube is nearly harmonic, with trapping frequency $\omega_{⊥}/2\pi \approx 33.9\;\text{kHz}$ . After loading in the 1D tubes, the maximal temperature is T≃38nK ( $k_{B}T/2\pi \hbar ≃0.79\;\text{kHz}$ ) and the maximum chemical potential is μ/2πℏ≃1.7kHz , both estimated by comparison to QMC calculations, see Supplementary Materials, section 3. It very well satisfies the quasi-1D condition, $k_{B}T,\mu \ll \hbar \omega_{⊥}$ , with *k*_B_ the Boltzmann constant, for all results presented here below. Moreover, on top of the strong transverse confinement, the Gaussian-shaped lattice laser beams create an axial harmonic confinement along the tubes with frequency $\omega_{x}/2\pi \approx 84\;\text{Hz}$ . For ^87^Rb atoms ( $a_{3D}\approx 5.3\;\text{nm}$ ), it yields to $a_{1D}\approx -5.8\times 10^{-7}\;m$ and γ in the range from 1 to 1.4. Except whenever mentioned, we use these parameters in the following.

### Two-stage expansion scheme

To realize a TOF insensitive to interactions in the axial direction *x*, we take advantage of the quasi-1D structure of the tubes. Switching off the confinement laser beams instantaneously would induce very fast expansion in the transverse (*y* and *z*) directions, driven by the transverse-confinement kinetic energy in a characteristic time $t_{⊥}∼1/\omega_{⊥}≃5\;\mathrm{\mu s}$ , and a much slower expansion in the axial direction (*x*), with a characteristic time $t_{x}∼1/\omega_{x}≃2\;\text{ms}$ . The transverse expansion by a factor b(t)>1 is associated with a sharp reduction of the effective 1D coupling constant $g_{1D}^{\text{eff}}=g_{1D}/b{\left(t\right)}^{2}$ , so that the effect of interactions along *x* becomes negligible during the axial expansion stage. In practice, we use a controlled exponential ramp to switch off the confinement progressively in a time Δ*t* and then let the gas expand for a longer time $t_{\text{TOF}}$ (see Fig. 1B). Setting the ramping time such that $t_{⊥}\ll \Delta t\ll t_{x}$ ensures that the transverse expansion is slowed down, while the switch-off remains nearly in-stantaneous as regards the axial expansion. In the experiment, we use Δ*t* = 50 μs, close to the geometric average of the transverse and axial characteristic times $t_{⊥}$ and $t_{x}$.

It remains to make sure that the transverse dilution is sufficient when the axial expansion starts, so that the latter is not substantially affected by interactions. Owing to the strong initial transverse confinement, the transverse expansion is that of a weakly interacting, 2D Bose gas, and may be treated in the mean-field approximation [see details and a complete derivation in Supplementary Materials, section 1]. For harmonic confinement, this expansion is strictly self-similar in 2D with a time-dependent expansion factor *b*(*t*), solution of the second-order differential equation $\ddot{b}+\omega_{⊥}^{2}\left(t\right)b=\omega_{⊥}^{2}\left(0\right)/b^{3}$ (*39*, *40*), where $\omega_{⊥}\left(t\right)$ is the time-dependent transverse harmonic trap frequency during [ 0<t<Δt and $\omega_{⊥}\left(t\right)>0$ ] and after [ $\Delta t<t<\Delta t+t_{\text{TOF}}$ and $\omega_{⊥}\left(t\right)=0$ ] the ramp. The transverse expansion does not affect the 1D density for it results from integration over the transverse directions, $n_{1D}\left(x\right)=\int \text{dydz}\;n_{3D}\left(x,y,z\right)$ . In contrast, after an expansion duration *t*, with $0<t<\Delta t+t_{\text{TOF}}$ , the effective 1D coupling constant has decreased by a factor of $1/b{\left(t\right)}^{2}$ , $g_{1D}\left(t\right)=g_{1D}/b{\left(t\right)}^{2}$ . For *b*(*t*) sufficiently large, the axial expansion stage is now that of a 1D gas in the weakly interacting regime. The mean-field interaction term is $g_{1D}\left(t\right)n_{1D}$ and affects the expansion up to a cutoff momentum $k_{c}$ such that $g_{1D}n_{1D}/b^{2}\left(1/10\omega_{x}\right)≲\hbar^{2}k_{c}^{2}/2m$ , where we set a conservative starting time of the axial expansion at $t=1/10\omega_{x}\ll 1/\omega_{x}$ . The universal $1/k^{4}$ tails are expected for $k≳1/|a_{1D}|$ (*11*, *19*). Using the expression $g_{1D}=-2\hbar^{2}/{\mathrm{ma}}_{1D}$ and replacing $k_{c}$ by $1/|a_{1D}|$ , we obtain the criterion

$$4|a_{1D}|n_{1D}≲b{\left(1/10\omega_{x}\right)}^{2}$$ (2)

such that the $1/k^{4}$ tails are not substantially affected by the residual interactions. Solving the equation for *b*(*t*) for the exponential ramp of the transverse confinement $\omega_{⊥}\left(t\right)$ used in the experiment, we checked that the condition (Eq. 2) is satisfied for all results presented hereafter. Specifically, we find $5.2≲4|a_{1D}|n_{1D}≲8$ and $b\left(1/10\omega_{x}\right)\approx 17.8$ in our experiments, which sufficiently fulfills the condition, see Supplementary Materials, section 1.

Moreover, our system benefits from using all-optical evaporative cooling. It helps us avoid the influence of impurities created by a magnetic trap during evaporative cooling, which can substantially affect the amplitude of the $1/k^{4}$ tails (*32*). The estimated maximum temperature, T≃38 nK, is also below the threshold temperature $T_{A}=\hbar^{2}n^{2}/{\mathrm{mk}}_{B}≃65$ nK, below which hole anomalies may be expected (*33*). Last, our experimental setup allows us to perform a TOF of $t_{\text{TOF}}=30$ ms. For the ^87^Rb atoms used in the experiment and the initial length of the tubes, L≲20μm , it satisfies the far-field condition $t_{\text{TOF}}\gg {\mathrm{mL}}^{2}/2\hbar$ so that the measured momentum distributions are unaffected by the initial spatial distribution of the atoms.

### Measurement of the contact

**Fig. 2.**
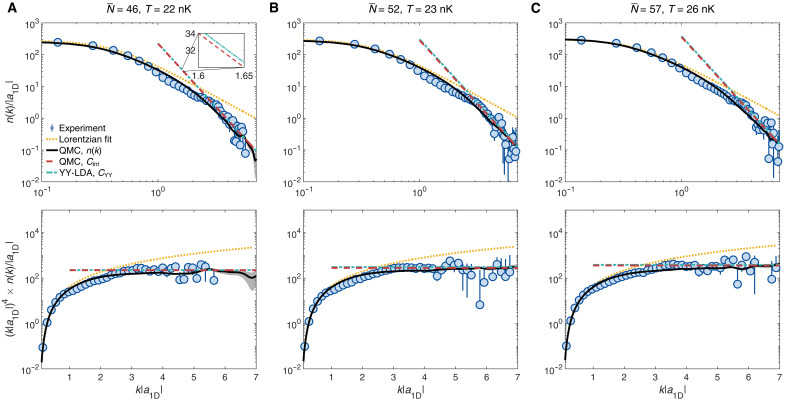
Momentum distributions for the trapped Lieb-Liniger gas. The top panels in (**A**), (**B**), and (**C**) show momentum distributions *n*(*k*) in log-log scale for various weighted average atom numbers $\bar{N}$ and temperatures *T*, indicated on top of each column. The bottom panels show the corresponding quantities $n\left(k\right)\times k^{4}$ in semilog scale for the same data. The figures display the experimental data (blue disks, with error bars corresponding to the SD), QMC results for the full momentum distribution (black solid line), Lorentzian fits to the low-*k* sector (orange dotted line), as well as the QMC estimates using the thermodynamic Tan relation (Eq. 3; red dashed line, $C_{\text{int}}$ ) and the YY-LDA estimates using the Tan sweep relation (Eq. 4; cyan dotted-dashed line, $C_{\text{YY}}$ ) estimates. Note that the $C_{\text{int}}$ and $C_{\text{YY}}$ are on top of each other and hardly distinguishable. The inset shows a magnified view of $C_{\text{int}}$ and $C_{\text{YY}}$.

Figure 2 shows three typical 1D momentum distributions, measured as discussed above, for various weighted average particle numbers $\bar{N}$ and temperatures *T* (indicated on top of the figure). The upper row shows the momentum distribution *n*(*k*) in log-log scale, while the lower row shows $n\left(k\right)\times k^{4}$ in semilog scale for the same data. In the experiment, the total atom number is found by integrating the full measured momentum distribution, and $\bar{N}$ determined as the weighted average over the tubes represents the relevant typical atom number (*41*–*45*), see Supplementary Materials, section 2.1. We have checked that the momentum distributions computed in QMC calculations either by averaging the contributions of all tubes with a distribution of atom numbers or using a single tube with $\bar{N}$ atoms yield very similar results in the parameter range considered in this work, see Supplementary Materials, section 2.2. To estimate the temperature in the experiment, we run several QMC calculations for a tube with the same weighted average atom number $\bar{N}$ and trapping frequency $\omega_{x}$ as in the experiment, and various temperatures. We then compare the momentum distribution found in the experiment to the ones found by the QMC calculations and identify the QMC result that best fits the experimental data in the low-*k* sector (typically $k≲1/|a_{1D}|$ ) and ignoring the tails. The temperature of this QMC calculation is the estimated temperature in the experiment, see Supplementary Materials, section 2.3. We then obtain good agreement between the experimental data (blue dots) and the QMC calculations (black solid line), not only in the fitted low-*k* sector but also in the tails with $k≳1/|a_{1D}|$ , see Fig. 2. In the low-*k* sector, both experimental and QMC results are consistent with a Lorentzian momentum distribution (orange dotted line) with half width at half maximum $\Delta k=\alpha k_{B}T/\hbar n_{0}$ with *T* the temperature estimated as above and $n_{0}$ the atom density at the center of the trap. The Lorentzian form of the low-*k* sector is due to the long-distance exponential decay of the one-body correlation function expected for a finite-temperature gas. The heuristic parameter α encapsulates the effect of axial trapping and finite interactions (*46*–*48*). We estimate it by fits of a Lorentzian function to the QMC data in the low-*k* sector, and we find α ≈ 0.8 for intermediate Lieb-Liniger parameter, γ ~ 1, relevant for our system, see Supplementary Materials, section 2.3. Note that, in this low-*k* sector, beyond Lorentzian behavior has been predicted in (*49*, *50*) and observed in (*51*). However, for the parameters of our experiment, these corrections are negligible, see Supplementary Materials, section 2.3.

We now focus on the large-*k* tails of the momentum distributions (typically $k≳4/|a_{1D}|$ ). There, the Lorentzian distribution breaks down and the data show algebraic tails, consistent with the expected universal behavior in $1/k^{4}$ . This is visible in the lower row of Fig. 2, which shows that the quantity $n\left(k\right)\times k^{4}$ approaches an asymptotic constant value. The latter is nothing but the Tan contact *C*. The experimental data for *n*(*k*) (blue dots) are then compared to three independent theoretical estimates. First, the comparison to the QMC momentum distribution (black solid line) confirms the good agreement with the experimental data in semilog scale. Second, we directly compute the Tan contact from QMC calculations using the thermodynamic Tan relation (*12*)

$$C_{\text{int}}=\frac{2g_{1D}m^{2}}{\hbar^{4}}\langle H_{\text{int}}\rangle$$ (3)

where $\langle H_{\text{int}}\rangle$ is the average interaction energy found numerically (red dashed line). Note that the calculation of the latter is independent of the momentum distribution also calculated using QMC, see Supplementary Materials, section 3. We, nevertheless, find good agreement between $C_{\text{int}}$ and the weight of the momentum tails found experimentally or in the QMC calculations. This confirms that the tails of the momentum distribution yield the Tan contact, which is thus accurately measured in the experiment. This provides a direct measurement of the Tan contact for an interacting Bose gas from the momentum distribution. Third, we also compare the experimental result to the contact computed using the Tan sweep relation (*9*)

$$C_{\text{YY}}=\frac{4m}{\hbar^{2}}{\left.\frac{\partial \Omega }{\partial a_{1D}}\right|}_{T,\mu }$$ (4)

(blue dashed line), where Ω is the grand potential, here calculated using Yang-Yang (YY) thermodynamics within local density approximation (LDA) (*21*). The YY-LDA estimate is in fair agreement with both experimental data and QMC results. Such consistency between all estimates is found for all the experiments presented in this work, corresponding to the temperature range 14nK<T<38nK and weighted average atom number $41<\bar{N}<75$.

### Thermodynamic properties of the Tan contact

**Fig. 3.**
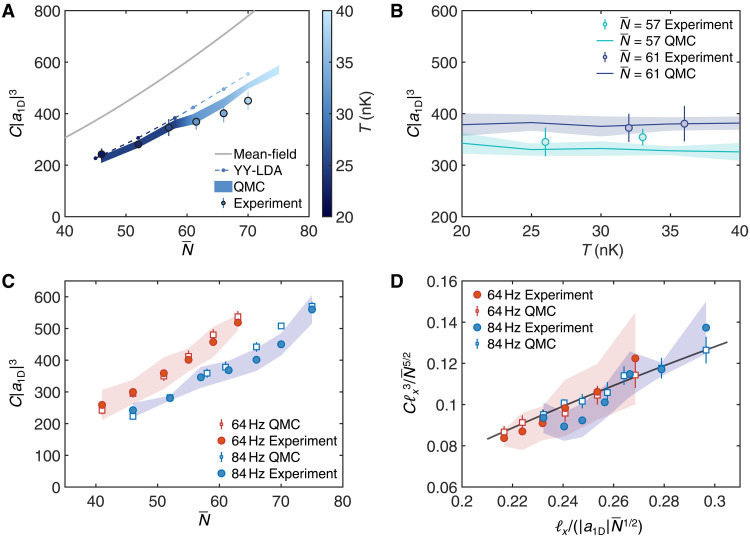
Tan’s contact versus atom number and temperature, and universal scaling. (**A**) Contact versus particle number $\bar{N}$ . The figure shows the experimental data (disks with error bars, where the shades of blue represent the temperature), the QMC results for $C_{\text{int}}$ (solid areas represent error bars, Eq. 3), the YY-LDA prediction (blue dashed line, Eq. 4), and the mean-field prediction (gray solid line, Eq. 5). (**B**) Contact versus temperature for $\bar{N}$ = 57 and 61. The disks with error bars represent experimental data, while the solid lines denote QMC results, with shaded areas indicating the corresponding QMC error bars. Within a specific temperature range, both datasets indicate that *C* remains nearly constant with temperature. (**C**) Contact versus particle number for two sets of data corresponding to $\omega_{x}/2\pi =64\;\text{Hz}$ (lattice amplitude *V* = 40 *E*_r_, red markers) and $\omega_{x}/2\pi =84\;\text{Hz}$ (*V* = 70 *E*_r_, blue markers). The squares are QMC results, while the disks are experimental data, and the shaded areas correspond to the experimental error bars. (**D**) Same data as in (C) but plotted according to the scaling of Eq. 6. The solid line is a fit to the QMC data.

In the experiment, the Tan contact *C* is extracted from fits to the asymptotic, large *k*, limit of the data for $n\left(k\right)\times k^{4}$ as in the lower row of Fig. 2. The results for various values of the weighted average particle number $\bar{N}$ are shown in Fig. 3A, where the particle number is tuned by controlling the parameters of the evaporative cooling process. At the same time, the final temperature *T* (encoded in the color scale) varies, typically between 22 and 38nK . However, it is expected that, in the considered regime $|a_{1D}|/\lambda_{\text{dB}}∼0.5$ , with $\lambda_{\text{dB}}=\sqrt{2\pi \hbar^{2}/{\mathrm{mk}}_{B}T}\approx 1.08\times 10^{-6}$ m the de Broglie wavelength at T=30nK and $|a_{1D}|\approx 5.8\times 10^{-7}$ m, the value of the contact *C* does not substantially depend on the temperature (*21*, *52*). To check this, we fixed the weighted average number of particles while varying the temperature. A representative result for $\bar{N}=57\;\text{and}\;61$ is shown in Fig. 3B. It confirms that, in the temperature range that we achieved here, the value of *C* extracted from the experiments (data points) is almost constant within about 10% and compatible with the theoretical prediction (solid lines). In the following, we can thus disregard the effect of temperature fluctuations on the contact *C*.

Consistently with theoretical predictions, we find that the contact increases monotonically with the particle number, see Fig. 3A. To understand the experimental data, we may first compare them with the mean-field prediction (*8*, *21*, *52*)

$$C=\frac{\eta N^{5/3}}{\ell_{x}^{4/3}{a_{1D}}^{5/3}}$$ (5)

where $\eta =4\times 3^{2/3}/5≃1.66$ and $\ell_{x}=\sqrt{\hbar /m\omega_{x}}$ is the axial harmonic oscillator natural length, shown as a gray solid line in Fig. 3A. We then find that our data strongly deviate from this simple estimate by more than 46% for all data points. We may now compare the experimental data to many-body calculations based on both QMC, Eq. 3 (blue solid line), and YY-LDA (blue dashed line). Clearly, the experimental data are in good agreement with the exact QMC calculations, within 12%. The YY-LDA is also in fair agreement with the experimental data, although with a larger deviation of about 21%, which we attribute to the moderate accuracy of LDA for a system the size of ours. The fact that the experimental data are consistent with exact many-body predictions but strongly differ from a mean-field calculation indicates strong beyond mean-field effects. This is consistent with the fact that the Lieb-Liniger parameter γ ranges from 1.0 to 1.4 in the experiment.

### Universal scaling of the Tan contact

Last, our experimental setup allows us to test the predicted universal two-parameter scaling of the Tan contact predicted in (*21*)

$$C=\frac{N^{5/2}}{\ell_{x}^{3}}f\left(\xi_{\gamma },\xi_{T}\right)$$ (6)

with $\xi_{\gamma }=-\ell_{x}/a_{1D}\sqrt{N}$ and $\xi_{T}=-a_{1D}/\lambda_{\text{dB}}$ . The interaction parameter $\xi_{\gamma }$ is the counterpart of the Lieb-Liniger parameter γ for a trapped system and $\xi_{T}$ accounts for thermal effects. Equation 6 originates from the combination of the two thermodynamic relations $C=\left(4m/\hbar^{2}\right){\partial \Omega /\partial a_{1D}|}_{T,\mu }$ and $N=-{\partial \Omega /\partial \mu |}_{T,a_{1D}}$ , assuming LDA. As noted above the latter is a fair, although not very accurate, approximation for our experimental setup. By varying the 2D lattice amplitude creating the tubes, we can tune the longitudinal trapping frequency $\omega_{x}$ and the coupling constant $g_{1D}$ or, equivalently, the parameters $\ell_{x}$ and $a_{1D}$ . We choose two values of the lattice potential depth: (i) *V* = 40 *E*_r_, which corresponds to $\omega_{x}/2\pi \approx 64\;\text{Hz}$ and $a_{1D}≃-0.78\;\mu m$ , and (ii) *V* = 70 *E*_r_, which corresponds to $\omega_{x}/2\pi \approx 84\;\text{Hz}$ and $a_{1D}≃-0.58\;\mu m$ . Plotting $C{|a_{1D}|}^{3}$ versus the particle number $\bar{N}$ , as in Fig. 3C, we observe two groups of data, corresponding to each set of parameters (*V* = 40 *E*_r_, red markers; *V* = 70 *E*_r_, blue markers), that are separated beyond their corresponding uncertainties (shaded areas). Again, the experimental (disks) and QMC (hollow squares) results are in good agreement. Then, plotting the same data using the rescaled quantities $\tilde{C}=C\ell_{x}^{3}/{\bar{N}}^{5/2}$ and $\xi_{\gamma }=-\ell_{x}/a_{1D}\sqrt{\bar{N}}$ , we observe data collapse within error bars, see Fig. 3D. This is consistent with the universal two-parameter scaling of Eq. 6. The black solid line in Fig. 3D is a fit to the QMC data and represents the universal scaling function *f* versus $\xi_{\gamma }$ . Because the temperature variation is negligible in the considered experimental conditions, the value of $\xi_{T}$ is fixed. Deviations of the rescaled experimental data to the QMC prediction are within 10%.

## DISCUSSION

Our work reports the first experimental measurement of the Tan contact *C* in a correlated Lieb-Liniger gas, from the direct observation of the large-momentum tails. Leveraging the quasi-1D structure of our system, we devised a two-stage expansion scheme sequence, which prevents detrimental effects of interactions during the gas expansion. Comparison of the observed short-*k* sector of the momentum distribution with QMC calculations allows us to determine the experimental temperature, while the weight of the large-momentum tails yields a direct measure of *C*. Good agreement is found with theoretical predictions, and we show clear beyond mean-field effects. Varying the particle number, the temperature, as well as the interaction strength via transverse confinement, allowed us to realize a first test of the universal two-parameter scaling law predicted in (*21*).

Direct measurement of the Tan contact as realized here provides a wealth of information about the thermodynamics of the Lieb-Liniger gas. The accessible ranges of atom number and temperature are presently limited in our system, but this can be improved by adapting the laser cooling and evaporative stages of the atomic gas preparation. Using stronger transverse confinement can also be used to access stronger interaction regimes. It would allow for systematic demonstration of the two-parameter scaling law, Eq. 6. In this respect, using a lower longitudinal trapping would be beneficial for better realizing the LDA condition used to derive Eq. 6. It would, for instance, pave the way to observation of fermionization of the strongly interacting Lieb-Liniger gas at finite temperatures, which is marked by a characteristic maximum of the rescaled Tan contact (*21*). Our approach may also be extended to further quantum models in confined geometries, for instance, ultracold Fermi gases and Bose-Bose, Bose-Fermi, or Fermi-Fermi mixtures, the thermodynamics of which may also be characterized by the properties of the contact (*5*, *10*, *23*, *53*, *54*). While Feshbach resonances may be alternatively used to switch off interactions during TOF imaging in single-component gases, our approach has the advantage of being applicable also to mixtures.

## Supplementary Materials

### This PDF file includes:

Supplementary Text Figs. S1 to S3 References
