# Supplementary material for: Direct measurement of Tan’s contact in a one-dimensional Lieb-Liniger gas
Source: Sci Adv. 2025 Oct 3;11(40):eadv3727. doi: 10.1126/sciadv.adv3727 (PMC12494025; doi:10.1126/sciadv.adv3727)
Supplement: Supplementary file 1 — Supplementary Text Figs. S1 to S3 References [file sciadv.adv3727_sm.pdf]

Science Advances
AAAS

Supplementary Materials for

Direct measurement of Tan's contact in a one-dimensional Lieb-Liniger gas

Qi Huang *et al.*

Corresponding author: Hepeng Yao, hepeng.yao@pku.edu.cn; Xuzong Chen, xuzongchen@pku.edu.cn; Laurent Sanchez-Palencia, laurent.sanchez-palencia@polytechnique.edu

Sci. Adv. **11**, eadv3727 (2025)
DOI: 10.1126/sciadv.adv3727

This PDF file includes:

Supplementary Text
Figs. S1 to S3
References

1 Initial transverse dynamics in the two-stage expansion scheme

The transverse dynamics in the first stage of our two-stage expansion scheme is that of a weakly-interacting 2D Bose gas, owing to the strong initial transverse confinement. It is governed by the time-dependent Gross-Pitaevskii equation

$$i\hbar \frac{\partial \psi(\mathbf{r}, t)}{\partial t} = \left[\frac{-\hbar^2 \nabla^2}{2m} + \frac{m\omega_\perp^2(t)\mathbf{r}^2}{2} + g\,|\psi(\mathbf{r}, t)|^2 \right] \psi(\mathbf{r}, t), \tag{S1}$$

where g is the 2D coupling constant and $\psi(\mathbf{r}, t)$ is the transverse wavefunction with $\mathbf{r} = (y, z)$. For an arbitrary time-evolving trap frequency $\omega_\perp(t)$, the solution is self-similar and the atom density $n(\mathbf{r}, t) = |\psi(\mathbf{r}, t)|^2$ fulfills $n(\mathbf{r}, t) = b(t)^{-2} n\left[b(t)^{-1}\mathbf{r}, 0\right]$, where $b(t)$ is the expansion factor (*39,40*). Note that while this is correct only in the Thomas-Fermi regime in 3D, it is universal in 2D, that is irrespective of the interaction strength (*40*). The expansion factor $b(t)$ is then governed by the second-order differential equation

$$\ddot{b}(t) + \omega_\perp^2(t) b(t) = \frac{\omega_\perp^2(0)}{b^3(t)}. \tag{S2}$$

In the experiment, the transverse confinement is created by a 2D optical lattice of amplitude $V(t)$, and we have $\omega_\perp(t) = \hbar k^2 \sqrt{V(t)/E_\mathrm{r}}/m$.

For a sudden switch-off of the 2D optical lattice, we have $\omega_\perp(t) = 0$ for any $t > 0$, and Eq. S2 may be solve exactly (*39*),

$$b(t) = \sqrt{1 + [\omega_\perp(0)t]^2}. \tag{S3}$$

In our two-stage expansion scheme, we switch off the 2D optical lattice in a time Δt following an exponential ramp such that

$$\omega_\perp(t) = \omega_\perp(0) \left[\frac{\exp(-4t/\Delta t + 4) - 1}{\exp(4) - 1} \right]^{1/2} \tag{S4}$$

for $0 < t < \Delta t$ and $\omega_\perp(t) = 0$ for $t \geqslant \Delta t$. Equation S2 is then solved numerically. The result is shown in Fig. S1 and compared to Eq. S3, with $\Delta t = 50$ µs and a demonstration time ranging from 0 to $1/10\omega_x = 200$ µs. We find that the exponential ramp substantially slows down the transverse expansion and we find $b(1/10\omega_x) \simeq 17.8$.

2 Weighted average atom number and temperature estimate

Here, we discuss the estimates of the atom number distribution, the weighted average atom number, and the temperature of our system, as well as quantum Monte Carlo (QMC) simulations within a single tube with weighted average atom number.

2.1 Atom number distribution and weighted average atom number

At the beginning of the experiment, we obtain a 3D BEC in a nearly harmonic trap with frequencies $\Omega_x/2\pi \approx 23\,\text{Hz}$, $\Omega_y/2\pi \approx 20\,\text{Hz}$, $\Omega_z/2\pi \approx 28\,\text{Hz}$. For the large particle number in our experiment, we are in the Thomas-Fermi regime where the 3D Gross-Pitaevskii equation for the BEC wave function $\psi(\mathbf{r})$ reduces to

$$\left[V(\mathbf{r}) + g|\psi(\mathbf{r})|^2\right]\psi(\mathbf{r}) = \mu\psi(\mathbf{r}), \tag{S5}$$

where $V(\mathbf{r})$ is the harmonic trap potential, g is the 3D coupling constant, and μ is the chemical potential.

The 2D lattice is then ramped up, which cuts the system into 1D tubes. Since the loading process is slow, we can compute the atom number distribution using the rescaled coupling constant

$$\tilde{g} = g\frac{\pi(V_0/E_\mathrm{r})^{1/2}}{2\left(\mathrm{Erf}\left[\pi(V_0/E_\mathrm{r})^{1/4}/2\right]\right)^2}, \tag{S6}$$

where V_0 is the 2D lattice depth (*55*). Such a description includes the effect of the system expansion during the loading of lattice. The corresponding chemical potential is

$$\mu' = \frac{\hbar\bar{\omega}}{2}\left(15N\frac{a_\mathrm{s}}{\bar{\ell}}\frac{\tilde{g}}{g}\right)^{2/5}, \tag{S7}$$

where $\bar{\ell} = \sqrt{\hbar/m\bar{\omega}}$ with $\bar{\omega} = \sqrt{\omega_x\Omega'_y\Omega'_z}$ is the oscillation length and a_s is 3D s-wave scattering length. Note that the transverse trapping frequencies are slightly modified due to the Gaussian transverse shape of the lattice beams and are nearly equal, $\Omega'_y \approx \Omega'_z \approx \Omega'_\perp$. With these two values, we can estimate the Thomas-Fermi radius in the transverse directions

$$R_\mathrm{TF} = \sqrt{\frac{2\mu'}{m\Omega'^2_\perp}}, \tag{S8}$$

and the atom number distribution,

$$N_{i,j} = N_{0,0}\left[1 - \frac{i^2 + j^2}{(R_\mathrm{TF}/d)^2}\right]^{3/2}, \tag{S9}$$

where i, j are the index numbers along the y and z directions, d is the lattice spacing, and $N_{0,0}$ is the number of atoms in the central tube. The value of $N_{0,0}$ can be deduced from the total atom number N with the condition $N = \sum_{i,j} N_{i,j}$, which yields

$$N_{0,0} = \frac{5Nd^2}{2\pi R_{\mathrm{TF}}^2}, \tag{S10}$$

and the weighted average atom number is then

$$\overline{N} = \sum_{i,j} \frac{N_{i,j}^2}{N}. \tag{S11}$$

The Lieb-Liniger parameter is estimated according to the averaged density $\bar{n} = \overline{N}/L_{\overline{N}}$ with $L_{\overline{N}}$ the tube length computed from the QMC calculations, $\gamma = mg_{1\mathrm{D}}/\hbar^2\bar{n}$.

2.2 Single-tube QMC calculations with weighted average atom number

When comparing our experimental data to QMC calculations in the main text, we always simulate our system by one single tube with a number of atoms equal to the weighted average atom number $\overline{N}$ as estimated above, instead of simulating all tubes. A similar approach has been successfully used in previous experiments, see for instance Refs. (*41–44*). Here, we check this approximation and show that it is very accurate over the parameter range considered in this work.

Figure S2 shows a comparison of momentum distributions for six typical cases. The upper row shows the three cases in Fig. 2 of the main text, namely (A) $\overline{N} = 46$ and $T = 22\,\mathrm{nK}$, (B) $\overline{N} = 52$ and $T = 23\,\mathrm{nK}$, and (C) $\overline{N} = 57$ and $T = 26\,\mathrm{nK}$. The lower row shows three additional cases (see detailed parameters in the Figure caption), which covers the minimum and maximum atom number data for the two curves in Fig. 3 of the main text. In all these subplots, we simulate all tubes with the atom distribution of Eq. S9 (solid black line) or one single tube with the weighted average number of atoms of Eq. S11 (dashed cyan line). Clearly, they show very good agreement for both low-k and large-k regimes. In particular, they show very similar $1/k^4$ tails with amplitudes (contact C) differing by less 10%.

2.3 Temperature

The temperature in the experiment is estimated by comparing the low-k sector of the momentum distributions obtained in the experiment and the QMC calculations. In practice, we follow the

thermometry procedure also used in Refs. (*45, 56*). We run QMC simulations with all parameters identical to the experiment (same particle number, interaction strength, and trapping frequency) but various temperatures. We then identify the temperature of the experiment as the temperature of the QMC calculation that best matches the experiment. Figure S3 shows an example of the 1D momentum distribution found in the experiment and the corresponding QMC calculation which fits the latter. In this example, which corresponds to the case of Fig. 2C in the main text, the temperature of this QMC simulation is $T = 26\,\mathrm{nK}$.

The long-distance one-body correlation function is dominated by thermal fluctuations and decays exponentially. The low-k momentum distribution is the Fourier transform of this exponential decay, i.e. a Lorentzian function, which is sensitive to the temperature. For a weakly-interacting, homogeneous 1D Bose gas, the HWHM of the low-k Lorentzian function is $\Delta k = 1/L_\phi$ with L_ϕ the coherence length (*57*). In the presence of a harmonic trap, it has been found that it still holds but with a modified HWHM,

$$\Delta k = \frac{\alpha}{L_\phi},\ L_\phi = \frac{\hbar^2 n_0}{m k_\mathrm{B} T} \tag{S12}$$

where n_0 is the central particle density, and α is a phenomenological correction factor, which depends on the interaction strength (*46–49*). Here we find that the same holds for the Lieb-Liniger gas with stronger interactions for a parameter $\alpha \simeq 0.8$ fitted from QMC calculations.

Note that, in this work, only the low-k sector is used for thermometry and the large-k sector is used to observe and measure the contact. Both regimes are expected from the general properties of the Lieb-Liniger gas. More precisely, a three-regime structure is generally expected (*58, 59*). For a homogeneous system, the short-distance (large k) regime dominated by the $1/k^4$ Tan tail is expected for $k \gtrsim k_c$ where $k_c = \sqrt{m g_{1\mathrm{D}} n}/\hbar$, while the long-distance (low k) regime dominated by thermal fluctuations is expected for $k \lesssim k_T$ where $k_T = 2\pi m k_\mathrm{B} T/\hbar^2 n$. The intermediate regime is dominated by Luttinger liquid behavior, characterized by algebraic decay of correlation functions. For typical parameters in our experiment, we have $T = 26\,\mathrm{nK}$ and $n_0 = 5.3 \times 10^{-6}\,\mathrm{m}^{-1}$ (parameters of Fig. 2C in the main paper). It leads to $k_T \simeq 3.60|a_{1\mathrm{D}}|^{-1}$ and $k_c \simeq 3.15|a_{1\mathrm{D}}|^{-1}$. Therefore, for our work, the intermediate Luttinger regime cannot be observed, and we only observe the Lorentzian and Tan regimes in our momentum distributions.

3 Quantum Monte Carlo simulations

In the QMC calculations, we simulate the exact Hamiltonian (Eq. 1). The QMC is formulated in the path-integral representation within grand-canonical ensemble and uses updates exploiting the worm algorithm, similarly as in previous works (*21, 44*). In the experiment, the quasi-1D condition is very well satisfied and we simulate the system in strictly 1D geometry. For the QMC results presented in the manuscript, we consider a single tube with the weighted average atom number, but we also performed calculations for a bunch of tubes with the atom distribution estimated as discussed in section 2.1 to perform checks, see section 2.2. Thanks to imaginary-time space discretization, we can describe the harmonically-trapped system in continuous space. For a given temperature T, trap frequency ω, coupling constant $g_{1\mathrm{D}}$, and chemical potential μ, we can compute standard quantities such as the atom number and the interaction energy H_{int} of the system from the statistics of world lines. Then, the Tan contact can be extracted from the sweep relation Eq. 3. Moreover, the worm algorithm implementation also allows us to calculate the momentum distribution $n(k)$ as follows: We first compute the one-body correlation function $\langle\hat{\psi}^\dagger(x)\hat{\psi}(x')\rangle$ exploiting the open world line configurations (so-called G sector). We then perform a Fourier transform to obtain the momentum distribution,

$$n(k) = \frac{1}{L}\int \mathrm{d}x\mathrm{d}x' \,\langle\hat{\psi}^\dagger(x)\hat{\psi}(x')\rangle e^{ik\cdot(x-x')}. \tag{S13}$$

From the statistics of a large number of QMC configurations, we are able to compute $n(k)$ up to large values of k with small errorbars. It allows us to observe $1/k^4$ tails and extract the contact from their weight.

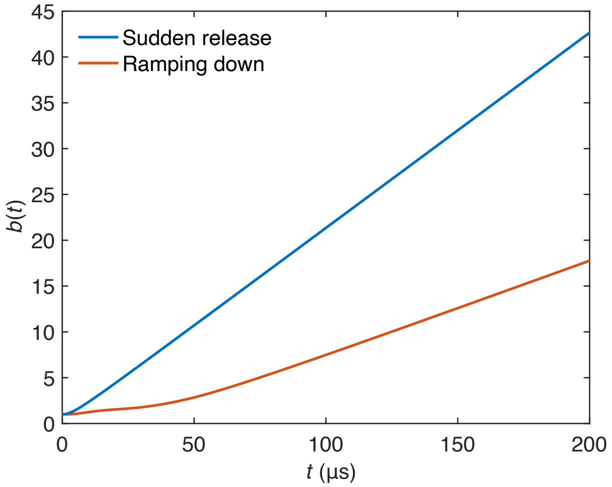

Figure S1: **Time evolution of the expansion parameter** $b(t)$**.** The blue line corresponds to sudden release, while the orange line represents the result of exponential ramping down.

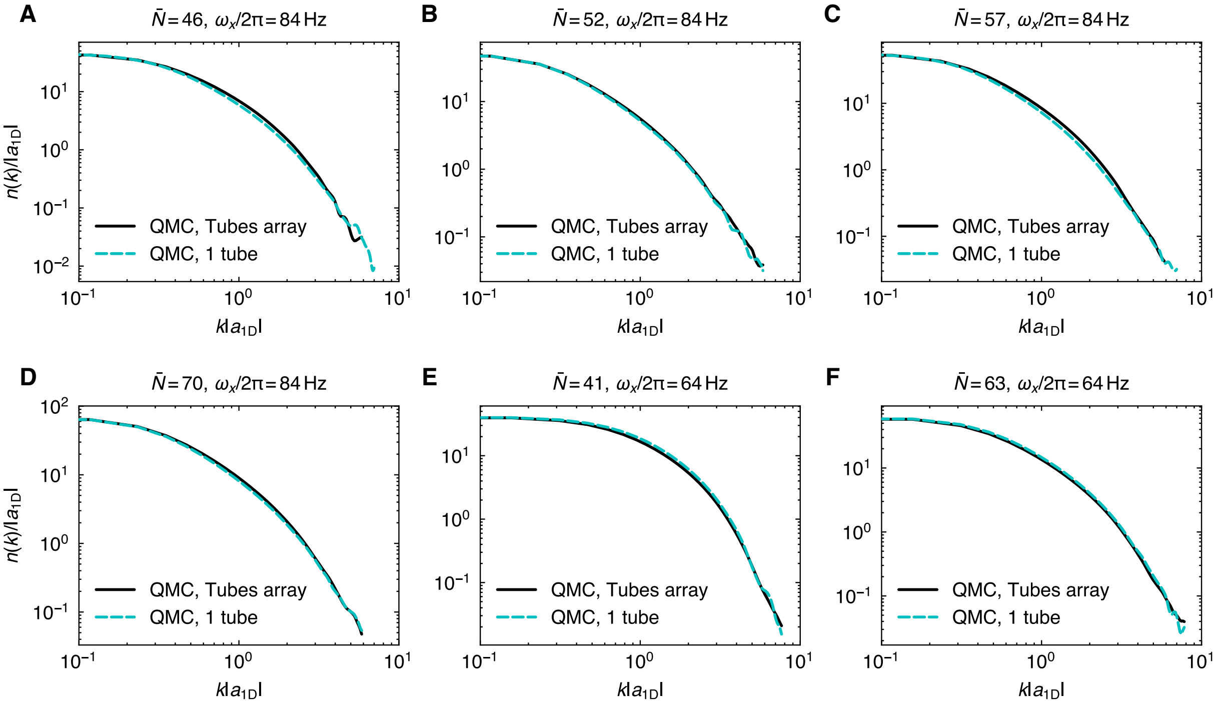

Figure S2: **Comparison of momentum distributions** $n(k)$ **computed with QMC for the full tube distribution (solid black line) and a single tube with weighted average atom number (dashed cyan line).** (**A**), (**B**) and (**C**) correspond to those of Fig. 2B of the main text, while (**D**), (**E**) and (**F**) show data from three points of Fig. 3C. These data cover the parameter ranges used in this work for both trapping frequencies $\omega_x/2\pi \approx 84\,\text{Hz}$ and $\omega_x/2\pi \approx 64\,\text{Hz}$.

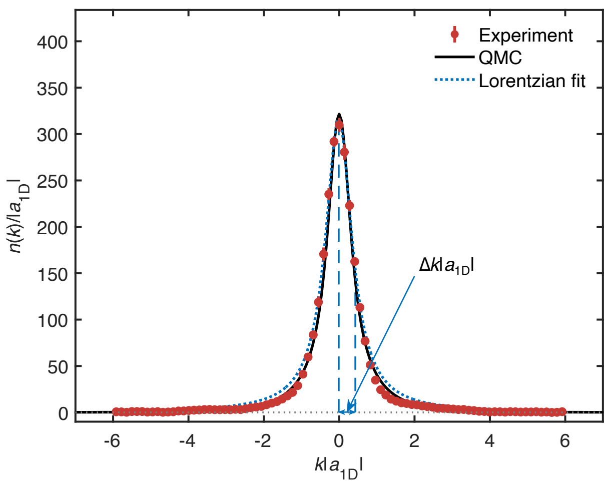

Figure S3: **Determination of temperature via momentum distribution matching.** By comparing the momentum distributions $n(k)$ from experimental data (red dots) with QMC calculations at different temperatures, we find the one (solid black line) that fits the profile to determine the temperature. A Lorentzian fit to the experimental low-momentum distribution is also shown (dotted blue line), along with its HWHM Δk. In this example, the weighted average atom number is $\overline{N} \approx 57$ and the trapping frequency is $\omega_x/2\pi \approx 84$ Hz.

REFERENCES AND NOTES

1. S. Sachdev, *Quantum Phase Transitions* (Cambridge Univ. Press, 2011).

2. H. Bruus, K. Flensberg, *Many-Body Quantum Theory in Condensed Matter Physics* (Oxford Univ. Press, 2004).

3. A. M. Tsvelik, *Quantum Field Theory in Condensed Matter Physics* (Cambridge Univ. Press, 2007).

4. M. Lewenstein, A. Sanpera, V. Ahufinger, B. Damski, A. Sen, U. Sen, Ultracold atomic gases in optical lattices: Mimicking condensed matter physics and beyond. *Adv. Phys.* **56**, 243–379 (2007).

5. I. Bloch, J. Dalibard, W. Zwerger, Many-body physics with ultracold gases. *Rev. Mod. Phys.* **80**, 885–964 (2008).

6. C. Chin, R. Grimm, P. Julienne, E. Tiesinga, Feshbach resonances in ultracold gases. *Rev. Mod. Phys.* **82**, 1225–1286 (2010).

7. V. E. Korepin, N. M. Bogoliubov, A. G. Izergin, *Quantum Inverse Scattering Method and Correlation Functions* (Cambridge Univ. Press, 1997).

8. M. Olshanii, V. Dunjko, Short-distance correlation properties of the Lieb-Liniger system and momentum distributions of trapped one-dimensional atomic gases. *Phys. Rev. Lett.* **91**, 090401 (2003).

9. S. Tan, Large momentum part of a strongly correlated Fermi gas. *Ann. Phys.* **323**, 2971–2986 (2008).

10. Y.-Q. Zou, B. Bakkali-Hassani, C. Maury, É. Le Cerf, S. Nascimbene, J. Dalibard, J. Beugnon, Tan's two-body contact across the superfluid transition of a planar Bose gas. *Nat. Commun.* **12**, 760 (2021).

11. S. Tan, Generalized virial theorem and pressure relation for a strongly correlated Fermi gas. *Ann. Phys.* **323**, 2987–2990 (2008).

12. S. Tan, Energetics of a strongly correlated Fermi gas. *Ann. Phys.* **323**, 2952–2970 (2008).

13. A. Minguzzi, P. Vignolo, M. P. Tosi, High-momentum tail in the Tonks gas under harmonic confinement. *Phys. Lett. A* **294**, 222–226 (2002).

14. D. M. Gangardt, G. V. Shlyapnikov, Stability and phase coherence of trapped 1D Bose gases. *Phys. Rev. Lett.* **90**, 010401 (2003).

15. M. A. Cazalilla, One-dimensional optical lattices and impenetrable bosons. *Phys. Rev. A* **67**, 053606 (2003).

16. M. Barth, W. Zwerger, Tan relations in one dimension. *Ann. Phys.* **326**, 2544–2565 (2011).

17. P. Vignolo, A. Minguzzi, Universal contact for a Tonks-Girardeau gas at finite temperature. *Phys. Rev. Lett.* **110**, 020403 (2013).

18. Y.-Y. Chen, Y.-Z. Jiang, X.-W. Guan, Q. Zhou, Critical behaviours of contact near phase transitions. *Nat. Commun.* **5**, 5140 (2014).

19. W. Xu, M. Rigol, Universal scaling of density and momentum distributions in Lieb-Liniger gases. *Phys. Rev. A* **92**, 063623 (2015).

20. E. Nandani, R. A. Römer, S. Tan, X.-W. Guan, Higher-order local and non-local correlations for 1D strongly interacting Bose gas. *New J. Phys.* **18**, 055014 (2016).

21. H. Yao, D. Clément, A. Minguzzi, P. Vignolo, L. Sanchez-Palencia, Tan's contact for trapped Lieb-Liniger bosons at finite temperature. *Phys. Rev. Lett.* **121**, 220402 (2018).

22. J. Decamp, M. Albert, P. Vignolo, Tan's contact in a cigar-shaped dilute Bose gas. *Phys. Rev. A* **97**, 033611 (2018).

23. O. I. Pâţţu, A. Klümper, A. Foerster, Universality and quantum criticality of the one-dimensional spinor Bose gas. *Phys. Rev. Lett.* **120**, 243402 (2018).

24. J. T. Stewart, J. P. Gaebler, T. E. Drake, D. S. Jin, Verification of universal relations in a strongly interacting Fermi gas. *Phys. Rev. Lett.* **104**, 235301 (2010).

25. E. D. Kuhnle, H. Hu, X.-J. Liu, P. Dyke, M. Mark, P. D. Drummond, P. Hannaford, C. J. Vale, Universal behavior of pair correlations in a strongly interacting Fermi gas. *Phys. Rev. Lett.* **105**, 070402 (2010).

26. R. J. Wild, P. Makotyn, J. M. Pino, E. A. Cornell, D. S. Jin, Measurements of Tan's contact in an atomic Bose-Einstein condensate. *Phys. Rev. Lett.* **108**, 145305 (2012).

27. S. Laurent, M. Pierce, M. Delehaye, T. Yefsah, F. Chevy, C. Salomon, Connecting few-body inelastic decay to quantum correlations in a many-body system: A weakly coupled impurity in a resonant Fermi gas. *Phys. Rev. Lett.* **118**, 103403 (2017).

28. B. Mukherjee, P. B. Patel, Z. Yan, R. J. Fletcher, J. Struck, M. W. Zwierlein, Spectral response and contact of the unitary Fermi gas. *Phys. Rev. Lett.* **122**, 203402 (2019).

29. C. Carcy, S. Hoinka, M. G. Lingham, P. Dyke, C. C. N. Kuhn, H. Hu, C. J. Vale, Contact and sum rules in a near-uniform Fermi gas at unitarity. *Phys. Rev. Lett.* **122**, 203401 (2019).

30. C. Qu, L. P. Pitaevskii, S. Stringari, Expansion of harmonically trapped interacting particles and time dependence of the contact. *Phys. Rev. A* **94**, 063635 (2016).

31. R. Chang, Q. Bouton, H. Cayla, C. Qu, A. Aspect, C. I. Westbrook, D. Clément, Momentum-resolved observation of thermal and quantum depletion in a Bose gas. *Phys. Rev. Lett.* **117**, 235303 (2016).

32. H. Cayla, P. Massignan, T. Giamarchi, A. Aspect, C. I. Westbrook, D. Clément, Observation of $1/k^4$-tails after expansion of Bose-Einstein condensates with impurities. *Phys. Rev. Lett.* **130**, 153401 (2023).

33. G. De Rosi, G. E. Astrakharchik, M. Olshanii, J. Boronat, Thermal fading of the$1/k^4$ tail of the momentum distribution induced by the hole anomaly. *Phys. Rev. A* **109**, L031302 (2024).

34. M. Olshanii, Atomic scattering in the presence of an external confinement and a gas of impenetrable bosons. *Phys. Rev. Lett.* **81**, 938–941 (1998).

35. D. S. Petrov, G. V. Shlyapnikov, J. T. M. Walraven, Regimes of quantum degeneracy in trapped 1D gases. *Phys. Rev. Lett.* **85**, 3745–3749 (2000).

36. E. H. Lieb, W. Liniger, Exact analysis of an interacting Bose gas. I. The general solution and the ground state. *Phys. Rev.* **130**, 1605–1616 (1963).

37. E. H. Lieb, Exact analysis of an interacting Bose gas. II. The excitation spectrum. *Phys. Rev.* **130**, 1616–1624 (1963).

38. M. Girardeau, Relationship between systems of impenetrable bosons and fermions in one dimension. *J. Math. Phys.* **1**, 516–523 (1960).

39. Y. Castin, R. Dum, Bose-Einstein condensates in time dependent traps. *Phys. Rev. Lett.* **77**, 5315–5319 (1996).

40. Y. Kagan, E. L. Surkov, G. V. Shlyapnikov, Evolution of a Bose-condensed gas under variations of the confining potential. *Phys. Rev. A* **54**, R1753–R1756 (1996).

41. F. Meinert, M. Panfil, M. J. Mark, K. Lauber, J.-S. Caux, H.-C. Nägerl, Probing the excitations of a Lieb-Liniger gas from weak to strong coupling. *Phys. Rev. Lett.* **115**, 085301 (2015).

42. N. Fabbri, M. Panfil, D. Clément, L. Fallani, M. Inguscio, C. Fort, J.-S. Caux, Dynamical structure factor of one-dimensional Bose gases: Experimental signatures of beyond-Luttinger-liquid physics. *Phys. Rev. A* **91**, 043617 (2015).

43. K.-Y. Li, Y. Zhang, K. Yang, K.-Y. Lin, S. Gopalakrishnan, M. Rigol, B. L. Lev, Rapidity and momentum distributions of one-dimensional dipolar quantum gases. *Phys. Rev. A* **107**, L061302 (2023).

44. Y. Guo, H. Yao, S. Ramanjanappa, S. Dhar, M. Horvath, L. Pizzino, T. Giamarchi, M. Landini, H.-C. Nägerl, Observation of the 2D-1D crossover in strongly interacting ultracold bosons. *Nat. Phys.* **20**, 934–938 (2024).

45. Y. Guo, H. Yao, S. Dhar, L. Pizzino, M. Horvath, T. Giamarchi, M. Landini, H.-C. Nägerl, Anomalous cooling of bosons by dimensional reduction. *Sci. Adv.* **10**, eadk6870 (2024).

46. F. Gerbier, J. H. Thywissen, S. Richard, M. Hugbart, P. Bouyer, A. Aspect, Momentum distribution and correlation function of quasicondensates in elongated traps. *Phys. Rev. A* **67**, 051602 (2003).

47. S. Richard, F. Gerbier, J. H. Thywissen, M. Hugbart, P. Bouyer, A. Aspect, Momentum spectroscopy of 1D phase fluctuations in Bose-Einstein condensates. *Phys. Rev. Lett.* **91**, 010405 (2003).

48. N. Fabbri, D. Clément, L. Fallani, C. Fort, M. Inguscio, Momentum-resolved study of an array of one-dimensional strongly phase-fluctuating Bose gases. *Phys. Rev. A* **83**, 031604 (2011).

49. M. A. Cazalilla, Bosonizing one-dimensional cold atomic gases. *J. Phys. B: At. Mol. Opt. Phys.* **37**, S1–S47 (2004).

50. S. Cheng, Y.-Y. Chen, X.-W. Guan, W.-L. Yang, R. Mondaini, H.-Q. Lin, Exact spectral function of one-dimensional Bose Gases. arXiv:2209.15221 [cond-mat.quant-gas] (2022).

51. B. Yang, Y.-Y. Chen, Y.-G. Zheng, H. Sun, H.-N. Dai, X.-W. Guan, Z.-S. Yuan, J.-W. Pan, Quantum criticality and the Tomonaga-Luttinger liquid in one-dimensional Bose gases. *Phys. Rev. Lett.* **119**, 165701 (2017).

52. K. V. Kheruntsyan, D. M. Gangardt, P. D. Drummond, G. V. Shlyapnikov, Pair correlations in a finite-temperature 1D Bose gas. *Phys. Rev. Lett.* **91**, 040403 (2003).

53. Y. Sagi, T. E. Drake, R. Paudel, D. S. Jin, Measurement of the homogeneous contact of a unitary Fermi gas. *Phys. Rev. Lett.* **109**, 220402 (2012).

54. J. Decamp, J. Jünemann, M. Albert, M. Rizzi, A. Minguzzi, P. Vignolo, High-momentum tails as magnetic-structure probes for strongly correlated SU(κ) fermionic mixtures in one-dimensional traps. *Phys. Rev. A* **94**, 053614 (2016).

55. M. Krämer, L. Pitaevskii, S. Stringari, Macroscopic dynamics of a trapped Bose-Einstein condensate in the presence of 1D and 2D optical lattices. *Phys. Rev. Lett.* **88**, 180404 (2002).

56. Y. Zhao, Y. Tian, J. Ye, Y. Wu, Z. Zhao, Z. Chi, T. Tian, H. Yao, J. Hu, Y. Chen, W. Chen, Universal dissipative dynamics in strongly correlated quantum gases. *Nat. Phys.* **21**, 530–535 (2025).

57. J. W. Kane, L. P. Kadanoff, Long-range order in superfluid helium. *Phys. Rev.* **155**, 80–83 (1967).

58. M. A. Cazalilla, R. Citro, T. Giamarchi, E. Orignac, M. Rigol, One dimensional bosons: From condensed matter systems to ultracold gases. *Rev. Mod. Phys.* **83**, 1405–1466 (2011).

59. T. Giamarchi, *Quantum Physics in One Dimension* (Clarendon Press, 2003), vol. 121.
